# Differences in the Factor Structure of the Eating Attitude Test-26 (EAT-26) in Different Cultures in Israel: Jews, Muslims, and Christians

**DOI:** 10.3390/nu13061899

**Published:** 2021-05-31

**Authors:** Zohar Spivak-Lavi, Ora Peleg, Orna Tzischinsky, Daniel Stein, Yael Latzer

**Affiliations:** 1 Department of Social Work, Max Stern, Yezreel Valley College, Yezreel Valley 1930600, Israel; 2 Education and School Counseling Departments, Max Stern, Yezreel Valley College, Yezreel Valley 1930600, Israel; orap@yvc.ac.il (O.P.); orna@yvc.ac.il (O.T.); 3 Department of Behavioral Science, Max Stern, Yezreel Valley College, Yezreel Valley 1930600, Israel; 4 Sackler Faculty of Medicine, Tel Aviv University, Tel Aviv-Yafo 6997801, Israel; prof.daniel.stein@gmail.com; 5 Chaim Sheba Medical Center, Medical School, Tel Hashomer 5262000, Israel; 6 School of Social Work, Faculty of Social Welfare and Health Sciences, University of Haifa, Haifa 3498838, Israel; ylatzer@univ.haifa.ac.il; 7 Rambam Medical Center, Eating Disorders Institution, Haifa 3109601, Israel

**Keywords:** eating disorders, EAT-26, cross-cultural, factor analysis

## Abstract

Background: In recent years, there has been a shift in the clinical presentation and, hence, diagnostic definitions of eating disorders (EDs), reflected in a dramatic change in the diagnostic criteria of EDs in the DSM-5. The Eating Attitudes Test-26 (EAT-26) is currently considered an accepted instrument for community studies of EDs, although it features an inconsistent factorial structure in different cultures. Therefore, it is essential to investigate whether the EAT-26 can still be considered an adequate instrument for identifying the risk of developing EDs in different cultures. The aim of the present study was to examine the construct validity and internal consistency of the EAT-26. Method: The study used exploratory factor analysis (EFA) and confirmatory factor analysis (CFA) among different cultural populations in Israel. Results: Findings indicated different factors in different ethnic groups, most of which do not correspond with the original EAT-26 three-factor structure. Results: The analysis yielded two main factors among Israeli Jews, four main factors among Israeli Muslim Arabs, and three main factors among Israeli Christian Arabs. Conclusion: These findings shed light on cultural factors affecting perceptions of the EAT-26 items. This calls for a reconsideration of the generalization of the original three-factor structure of the questionnaire in different cultures.

## 1. Introduction

### 1.1. Eating Disorders

The rate of eating disorders (EDs) has been on the rise in many countries in the Western world, including Israel, in recent decades [1,2] The prevalence of EDs in Western countries is about 0.5–1% for anorexia nervosa (AN), 1–2% for bulimia nervosa (BN), and 1–3.5% for binge eating disorder (BED) [3].

EDs can impair physical and mental health and quality of life [4] and lead to high rates of mortality [5]. Therefore, early detection of EDs is essential for preventing complications and increasing recovery [6]. Indeed, in recent years, there has been a growing attempt to develop measures aimed at identifying behavioral and psychological characteristics indicating the risk of developing an ED [7].

### 1.2. Self-Reported Screening Tools for EDs

The severity of symptoms and risk of eating disorders were traditionally assessed using the following tools: The Eating Disorder Inventory (64–91 items, depending on the version); the Eating Disorder Examination, Screening Version [8,9]; the Eating Disorder Examination Questionnaire [9]; the SCOFF Questionnaire [10]; and the Eating Attitudes Test EAT; 26–40 items, [11,12].

The EAT was used extensively to study eating-related disturbances in community populations because of the multiple facets it assesses and its excellent psychometric properties. The first version, the EAT-40, included seven factors: Food preoccupation, drive for thinness and body image preoccupations, vomiting and laxative abuse, dieting, slow eating, covert eating, and perceived pressure to gain weight [13]. Answers were rated on a 6-point Likert scale, with a cutoff point of 30 differentiating between people with normal and disturbed eating.

The EAT-40 was found to be valid for differentiating between patients with AN and community-based controls [12]. Nonetheless, it yielded a high percentage of false–positive scores among potentially high-risk groups: 29% among ballet students and 27% among modeling students [12]. Despite these problems, the EAT-40 was considered an adequate screening tool for detecting groups at risk of developing EDs in the general population [7].

After a series of studies, the questionnaire was shortened to 26 items [11]. The shorter version, the EAT-26, consisted of three factors: Dieting, bulimia and food preoccupation, and oral control, related to self-control about eating and perceived pressure from others to gain weight. Answers are rated on a 6-point Likert scale, with a cutoff of 20 for total score. People receiving a score of 20 or higher are considered to show an overall pattern of disturbed eating. Both cutoff points (20 for the EAT-26 and 30 for the EAT-40) have been supported by studies conducted among clinical and nonclinical samples and may assist in identifying people at risk of developing an ED [14,15,16,17,18].

The EAT-26 was previously assessed in different ethnic groups [7,13]. and translated and adapted into many languages including Arabic [19], Japanese [20], Italian [21], and Hebrew [16]. The questionnaire was ultimately declared the screening instrument of choice by the National Eating Disorders Screening Program organized by the National Mental Illness Screening Project in 1999.

Although the EAT-26 is one of the most useful instruments for ED-related research and clinical purposes [22], a major challenge in its use is its inconsistent factorial structure. Garner and colleagues [11] suggested a three-factor model based on principal component analysis: A dieting factor characterized by avoidance of calorific foods and preoccupation with being thinner, a bulimia and food preoccupation factor, and an oral control factor. Attempts to replicate the same factors have not always been successful. Many studies in English-speaking countries have reported either three, four, or five factors, with the number of items ranging from 16 to 25 [7]. In non-English-speaking samples, four to six factors have been found [23]. Although the Hebrew version has been found to follow the original three- factor construct, a fourth factor, awareness of food preoccupation, has also been identified [16].

### 1.3. EDs from a Cross-Cultural Perspective: Israel as a Case Study

Culture is an important factor affecting the clinical presentation of an ED [24]. While EDs are most common in Western societies, recently, their prevalence in non-Western societies, e.g., Asian societies, has been gradually increasing [25,26]. This is due to the exposure to Western cultural norms, including the thin body ideal, resulting from globalization, worldwide broadcast media, and social media [27]. Nonetheless, EDs might be perceived and experienced differently within different ethnic groups, and typical features from one ethnic group cannot always be applied to another [28,29]. Accordingly, can the widely used EAT-26, originally designed and tested among Canadian women 45 years ago, be a sensitive and appropriate screening tool for non-Western populations in the 21st century? To examine this question, we chose Israel as a case study.

Israel is a multicultural society encompassing various ethnic and religious groups, including immigrants from many different countries [3]. Israel’s population comprises 6.7 million Jews (74%) and 1.9 million Arabs (22%), of whom 1.6 million (18%) are Muslim Arabs and 177,000 (2%) are Christian Arabs [30]. The Jewish Israeli majority generally represents a modern, Western-oriented society, with some exceptional religious subgroups; the Arab community in Israel is still in the process of transitioning from a more traditional society to a relatively modern and Westernized one [31].

Previous studies in Israel using the EAT-26 have indicated that a considerable minority of Israeli Jewish adolescents in nonclinical settings show abnormal eating attitudes and weight concerns [2,32,33,34,35]. Five studies examined disordered eating attitudes and behaviors among Arab adolescent girls in communal samples in Israel. Among Muslim Arab adolescents, the rates of maladaptive eating behaviors were similar to those of Jewish Israelis [3,36,37]. To the best of our knowledge, despite extensive research conducted around the world and in Israel, neither the Hebrew and the Arabic factors of the EAT-26 nor the measurement invariance across samples were examined.

In view of this inconsistency in the number of factors and items in the EAT-26 and in light of the differences between Arab and Jewish subjects in the EAT-26 scores, in eating habits and in their attitudes to thinness, the present study aimed to reevaluate the factorial structure of the EAT-26 and determine whether it functions similarly among different ethnic subgroups in Israel. We sought to examine how each of the cultural groups understands the EAT-26 Questionnaire. For this purpose, we examined its measurement invariance properties in two versions, Hebrew and Arabic, using a large sample of adolescents, young adults, and adults in three Israeli groups: Jews, Muslim Arabs, and Christian Arabs. This allowed us to explore metric and scalar invariance and sources of measurement variability that may influence the factor analysis results and interpretation of the EAT-26 in each ethnic group.

We assumed that each cultural group absorbs cultural messages regarding nutrition and appearance differently and would therefore interpret the items of the EAT-26 differently. Specifically, we hypothesized that Israeli Jews, who generally endorse Western norms, would show a factor analysis closest to the typical three-factor structure of the EAT-26 in Europe and North America. Israeli Muslim Arabs, the least likely to endorse Western norms, were expected to show an EAT-26 profile that would differ the most from the typical EAT-26 profile. Israeli Christian Arabs were expected to fall between the two other groups in their EAT-26 presentation.

## 2. Materials and Methods

### 2.1. Participants

The sample included 2614 Israeli participants. We excluded 36 Druze, 48 of other religions and 10 missing religion; thus, we had a total of 2540 participants, of whom 1807 were women. The median age of the participants was 20 (age range = 14–40), and 48.1% were younger than 20. Of these participants, 1165 (45.9%) were Jewish, 1085 (42.7%) were Arab Muslim, and 290 (11.4%) were Arab Christian. Of the participants with a known place of residence, 43.7% lived in a city, 41.2% lived in a village, and the remaining 15.1% lived in other dwelling types. Most of the participants were middle-income earners (69.0%); 8.3% were high-income earners and 22.7% were low-income earners. Of the 2609 participants with a known family status, 1881 (79.6%) were single, 459 (19.4%) were married, and the remaining 24 (1.0%) were divorced or widowed. Table 1 represents the statistical differences of background variables among the three groups.

### 2.2. Instruments

For this study, we used the Hebrew [38] and Arabic [37] translations of the EAT-26. The EAT-26 consists of three factors: Dieting, bulimia and food preoccupation, and oral control related to self-control about eating and perceived pressure from others to gain weight. Sample items include: “I feel extremely guilty after eating” (dieting); “I find myself preoccupied with food” (bulimia and food preoccupation); and “I avoid eating when I’m hungry” (oral control). This is an extensively used screening questionnaire for measuring eating-related preoccupations and behaviors in nonclinical populations. It includes 26 items scored on a 4-point Likert scale (0–3), with a possible total of 0–78 points. A score of 20 or higher is used to detect cases of disturbed eating attitudes [11]. The EAT-26 is considered to have good psychometric properties (total internal consistency: α = 0.86; dieting factor α = 80; bulimia and food preoccupation factor: α = 67; oral control factor: α = 56 [39]. In the present study, EFA and CFA of EAT-26 were reexamined.

### 2.3. Procedure

The study protocol was approved by two college-based institutional review boards. The two colleges, one Jewish and one Arab, are located in northern Israel. Ten schools (four Jewish and six Arab) in northern Israel were chosen for the assessment of adolescent participants after receiving consent from the Ministry of Education, the school principals, administrators, and teachers, and the adolescents’ parents. In each school, three classes each from Grades 10, 11, and 12 were selected. Informed consent was obtained from all participants involved in the study and from the adolescents’ parents. In addition, the questionnaires were given to undergraduate and graduate students at these two colleges. Ads were distributed, and students who responded to the ads filled out the questionnaires manually. Completion of the questionnaires was voluntary, and respondents were told that they could stop their participation at any point. All participants were assured of anonymity. Exclusion Criteria: Unhealthy participants, or participants diagnosed with any physical or emotional problems, such as learning disabilities or eating disorders.

### 2.4. Data Analysis

To estimate whether the structure of the EAT-26 can be replicated in a nonclinical Israeli sample, we first conducted a CFA. Next, to estimate whether Israeli Jews, Arab Muslims, and Arab Christians perceive EDs in the same way, we conducted factorial invariance models of the EAT-26. In the first model, we tested for configural invariance (i.e., pattern invariance) to assess whether relevant items similarly assessed each EAT-26 cluster across groups (i.e., whether the EAT-26 dieting, bulimia and food preoccupation, and oral control clusters featured the same items in the three groups).

A good model fit was expected to support configural invariance; model fit was estimated by the comparative fit index (CFI), Tucker-Lewis index (TLI), root mean square error of approximation (RMSEA), and standardized root mean square residual (SRMR). CFI and TLI > 0.90 and RMSEA and SRMR < 0.07 are considered acceptable.

We then tested metric invariance (i.e., weak invariance) to assess whether the constructs’ factor loadings were similar across the three groups. Attaining invariance of factor loadings suggests that the constructs have the same meaning to all participants across groups. Metric invariance is assessed by comparing the fit of the configural model with that of the metric invariance model; a nonsignificant chi-square test would support metric invariance. Of note, metric invariance is not sufficient to justify the comparison of group means.

Next, we tested scalar invariance (i.e., strong invariance), assessing whether items have the same intercepts. Noninvariance of intercepts may indicate potential measurement bias, suggesting that larger forces, such as cultural norms or developmental differences, are influencing the manner in which participants respond to items across groups. Attainment of scalar invariance justifies the comparison of group means. Scalar invariance is assessed by comparing the fit of the metric model with that of the scalar invariance model; a nonsignificant chi-square test would support scalar invariance.

All models were estimated using the MPlus 8.3 structural equation modeling package. Missing data (0.7%) were handled with the full information maximum likelihood method.

## 3. Results

### 3.1. Confirmatory Factor Analysis

In the CFA, we assessed the constructs of the EAT-26 among nonclinical Israeli participants. Thirteen items were loaded on the dieting cluster, six items on the bulimia and food preoccupation cluster, and seven items on the oral control cluster. The model showed acceptable fit to the observed data, χ^2^(183) = 2031.43, *p* < 0.001, CFI = 0.94, TLI = 0.90, RMSEA = 0.06, SRMR = 0.04, supporting the three-factor structure of the EAT-26 among nonclinical Israeli populations.

### 3.2. Configural Invariance

To assess whether similar items assessed the three EAT-26 constructs across the three groups (Israeli Jews, Arab Muslims, and Arab Christians), we conducted a configural invariance model. The model showed a poor fit to the observed data, χ^2^(598) = 3197.67, *p* < 0.001, CFI = 0.92, TLI = 0.87, RMSEA = 0.07, SRMR = 0.07, indicating that the EAT-26 dieting, bulimia and food preoccupation, and oral control clusters do not feature the same items across the three groups. Given the lack of acceptable configural invariance among the groups, we decided not to examine metric or scalar invariances.

We, instead, conducted EFAs separately for each group to better explore the EAT-26 factorial constructs in each population. First, we conducted parallel analysis (1000 samples and 95% confidence), Velicer’s minimum average partial (squared and fourth power), and comparison data (*n* = 20,000 of a finite population of comparison data and 100 samples) tests to examine the ideal number of factors in each population. The analyses indicated that the ideal number of factors is two for Israeli Jews, four for Arab Muslims, and three for Arab Christians (see Table 1, Table 2 and Table 3).

Next, we conducted EFAs with principal axis factoring as the extraction method (because all items were positively skewed) and promax as the rotation method. Specifically, the EFAs yielded two factors instead of three among nonclinical Israeli Jews: Combined binge–purge and restricting behaviors (12 items) and dieting behaviors and fear and preoccupation with food weight and shape (11 items). Three items were excluded due to low loadings (<0.40; items 13, 19 and 25; Table 2).

Among nonclinical Israeli Arab Muslims, four main factors emerged: Binge–purge, restricting, and dieting behaviors (8 items), preoccupation with food, weight, and shape (4 items), fear of loss of control over eating (4 items), and eating behavior related to social pressure (3 items). Seven items were excluded due to low loadings (<0.40; items 5, 6, 10, 15, 19, 22, and 25; Table 3).

Among nonclinical Israeli Arab Christians, two main factors emerged: Combined binge–purge and dieting behaviors (15 items) and preoccupation with food, weight, and shape (5 items). Six items were excluded due to low loadings (<0.40; items 2, 5, 6, 13, 15, and 25; Table 4).

## 4. Discussion

The EAT-26, although constructed almost four decades ago [11], is still considered a widely accepted screening instrument for measuring eating-related preoccupations and behaviors in nonclinical populations. The EAT-26 has been confirmed as a reliable and valid instrument in English [11] and translated and validated in many languages including Hebrew [38] and Arabic [19,40]. The EAT-26 consists of 26 items and three subscales: Dieting, bulimia and food preoccupation, and oral control.

In recent years, dramatic changes have occurred in what is considered “normal” with respect to attitudes toward eating, weight, and dieting behaviors [41]. There has been also an increase in the use of supposedly “healthy” behaviors, which include excessive exercise and rigid “healthy” dieting, traditionally regarded as medically problematic, among nonclinical populations [42]. This has occurred alongside greater preoccupation from a much younger age with eating [43], physical appearance, food [44], and body image [45]. Another change with respect to EDs is the greater transition in recent years from restricting to binge–purge behaviors [46] alongside the increasing use among nonclinical populations of self-induced vomiting as a means to weight reduction [47,48]. Moreover, in contrast to traditionally accepted definitions of a normal range BMI (20.0–24.9 kg/m^2^ [49]), a BMI of 18.5 kgs/m^2^ may currently be considered as acceptable [50], likely because it is present in many young women without illness. Altogether, these processes cast doubt on whether a relatively old questionnaire such as the EAT-26 can still serve as a tool for identifying groups at risk of developing EDs in current community studies. It is, in addition, important to examine whether the questionnaire is interpreted similarly in different cultures.

Accordingly, the aim of the present study was to assess whether the three-factor structure of the original EAT-26 questionnaire [11] could still be replicated in multicultural nonclinical Israeli samples from different cultures. For this purpose, the construct validity and internal consistency of the questionnaire were examined using EFA and CFA. We assumed that each cultural group would perceive eating attitudes and behaviors in a different way and would therefore interpret the EAT-26 items differently. We further hypothesized that Israeli Jewish participants, who tend to endorse Western norms, would show a factor configuration closest to the original three-factor structure of the EAT-26 in Europe and North America. Israeli Muslim Arabs, the least likely to endorse Western norms, were expected to show an EAT-26 profile that would differ the most from the typical EAT-26 profile. Israeli Christian Arabs were expected to fall somewhere between the two other groups in their EAT-26 presentation.

The first hypothesis was confirmed, with different EAT-26 factors found for each ethnic group. The second hypothesis was, however, not confirmed, as all three groups showed an EAT-26 profile that was different from the original three-factor construct. Among Israeli Jews, the EFA yielded two main factors (see Table 2): The first included items describing mainly binge–purge and restricting behaviors, and the second included items describing mainly dieting behaviors and fear and preoccupation with food, weight, and shape. Three items from the original questionnaire were excluded: “Other people think that I am too thin” (item 13); “I display self-control around food” (item 19); and “I enjoy trying new rich foods” (item 25).

Among nonclinical Israeli Muslim Arabs, four main factors emerged: Binge–purge, restricting, and dieting behaviors; preoccupation with food, weight, and shape; fear of loss of control overeating; and eating behavior related to social pressure (see Table 3). Seven items from the original questionnaire were excluded: “I cut my food into small pieces” (item 5); “I am aware of the calorie content of foods that I eat” (item 6); “I feel extremely guilty after eating” (item 10); “I take longer than others to eat my meals” (item 15); “I display self-control around food” (item 19); “I feel uncomfortable after eating sweets” (item 22); and “I enjoy trying new rich foods” (item 25).

Among nonclinical Israeli Christian Arabs, two main factors emerged: Combined binge–purge and dieting behaviors and preoccupation with food, weight, and shape (see Table 4). Six items from the original questionnaire were excluded: “I avoid eating when I am hungry” (item 2); “I cut my food into small pieces” (item 5); “I am aware of the calorie content of foods that I eat” (item 6); “Other people think that I am too thin” (item 13); “I take longer than others to eat my meals” (item 15); and “I enjoy trying new rich foods” (item 25). The findings show some similarity in the Jewish and Arab Christian groups in that analyses of both groups yielded two factors. Additionally, eating-related behaviors (restricting or binge–purge) were differentiated from preoccupation with food. The finding that binge–purge behaviors accompany restricting and dieting behaviors suggests that, in contrast to the original EAT-26, bulimia is not so differentiated from AN-like behaviors (bearing in mind that the clinical entity of BN was defined for the first time only three years earlier;). This behavioral overlap might be associated with the greater transition from restricting to binge–purge behaviors in recent years [46]. 

In contrast to the findings in the two other groups, the configuration of the EAT-26 in the Arab Muslim group was less differentiated, featuring four factors. This may relate to the fact that Middle Eastern Muslim countries in general [51] and Israeli Muslim Arabs in particular [52] are currently in a state of transition, undergoing rapid sociocultural changes with influences from two distinct cultural values. On one hand, they are exposed to Western eating-related norms. On the other hand, many still live in rural areas with their families of origin and maintain traditional values and habits, including a lesser inclination to the thin body ideal [51]. The finding that seven items of the original EAT-26 were not included in the Arab Muslim version (and five in the Arab Christian version), in contrast to only three items not included in the Hebrew version, may support the notion that the EAT-26 might be less accurate for more traditional Arab groups in Israel.

Our findings further show that item 25, regarding the enjoyment of new food, was excluded in all three cultural groups in Israel—suggesting that not enjoying unfamiliar food is not necessarily considered a maladaptive consummatory pattern among these populations. By the same token, items 5 and 6, relating to anorectic-like eating behaviors and calorie counting, respectively, were excluded in both Israeli Arab populations, suggesting that typical Western anorectic preoccupations and behaviors might not be as prevalent in more traditional Middle Eastern ED patients. Altogether, these findings raise doubt about the validity of the EAT-26 cutoff point of 20 to differentiate between patients with EDs and controls in Middle Eastern populations, although the EAT-26 has been found, overall, to distinguish Israeli patients with EDs from non-ED individuals in clinical samples [53].

The finding of four EAT-26 factors among Israeli Muslim Arabs partly resembles a previous study conducted in Qatar [54] with 2692 Muslim women. In this prior study, five EAT-26 related factors were identified, showing a lack of scalar invariance across languages and challenging the use of the questionnaire for screening purposes in Qatar. The researchers recommended developing a more culturally sensitive questionnaire that includes cultural and BMI-specific cutoff points as a screening instrument for disordered eating among traditional nonclinical Muslim populations.

The present results are in line with several previous studies indicating that the EAT-26 might have an inconsistent factorial structure with consequent differences in its subscales [55,56]. Rogoza and colleagues [56] suggested the likelihood of new factors in the EAT-26 that were not identified in the original questionnaire. Specifically, similar to our findings among Israeli Muslim Arabs, they identified a novel social pressure factor, which increased the risk of predisposition to an ED. Nasser [55] suggested that the validity of the EAT-26 dieting factor has been the most stable throughout the years, concluding that if the validity of the EAT-26 is limited to the dieting factor, it is likely that the questionnaire measures only dieting-rated tendencies. Khaled and colleagues [54] concluded that the lack of scalar invariance across language and BMI categories poses important challenges for the use of the EAT-26 for screening people with disturbed eating.

### Limitations, Directions for Future Research, and Conclusions

Several limitations of the present study should be considered. First, the sample covered a wide age range (14–40 years), and EDs may manifest differently at different ages. Future studies should examine the effect of age on findings regarding the EAT-26 in community samples. Second, the present study was performed with healthy participants and not among clinical populations. Future studies should examine whether similar cultural differences in the factorial structure of the EAT-26 also appear in ED populations. If so, this would considerably limit the clinical utility of the EAT-26 among traditional Middle Eastern populations. Third, it would be interesting to assess the EAT-26 among other cultural subgroups in Israel such as Druze, Bedouins, or the ultra-Orthodox Jewish population. Finally, we recommend the development of adapted and culturally sensitive versions of the EAT-26 that take into consideration the different presentations of ED-related symptoms in different cultures and recent changes occurring in ED presentation. It should be noted that this would not be the first time that a well-established questionnaire has undergone cross-cultural adaption. A good example is the Positive and Negative Affect Schedule [57]. This tool for the assessment of emotional and mood states has been cited in scholarly papers thousands of times over the years [58]. In an attempt to develop an adequate cross-cultural instrument, studies have been undertaken with participants from numerous nationalities and cultures, including countries that have never before appeared in the literature on affect. These studies have produced a new 10-item international screening tool known as the I-PANAS-SF [58] which has been found psychometrically acceptable in different cultural populations [59]

To summarize, our findings in different Israeli cultural subgroups support the notion that manifestations of EDs are very sensitive to cultural influences and present differently in different cultures [13,60]. At this point, it seems that the EAT-26 questionnaire still differentiates between ill and non-ill participants of the same culture, although usually with different cutoff points in different cultures. The questionnaire probably remains suitable for the identification of disturbed eating in nonclinical European and North American populations even today but with necessary adaptations to account for changes in the presentation of EDs in recent years. However, as this study has shown, this is possibly not the case regarding the identification of disturbed eating in non-Western community cohorts, particularly in the Middle East.

## Figures and Tables

**Table 1:** Statistical differences of backgrounds among three groups: Jews, Arab Muslims, and Arab Christians.

	Total Participants (*N* = 2540)	Jewish (*N* = 1165)	Muslim (*N* = 1085)	Christian (*N* = 290)	X^2^	*p*
Age	21.6 ± 6.7 (14–40)	23.5 ± 6.6 (14–40)	20.0 ± 6.3 (14–40)	19.7 ± 6.4 (14–40)	95.02 ^1^	0.001
Age group					217.92	0.001
<20	1222 (48.1)	376 (32.3)	672 (61.9)	174 (60.0)		
20–29	957 (37.7)	578 (49.6)	291 (26.8)	88 (30.3)		
≥30	361 (14.2)	211 (18.1)	122 (11.2)	28 (9.7)		
Gender					24.37	0.001
Male	729 (28.7)	288 (24.7)	329 (30.4)	112 (38.6)		
Female	1807 (71.3)	876 (75.3)	753 (69.6)	178 (61.4)		
Residence					704.05	0.001
City	924 (43.7)	499 (62.6)	329 (31.6)	96 (34.8)		
Village	872 (41.2)	52 (6.5)	670 (64.4)	150 (54.3)		
Other	318 (15.1)	246 (30.9)	42 (4.0)	30 (10.9)		
Social Economic Status					31.60	0.001
High	53 (8.3)	23 (7.7)	26 (9.6)	4 (5.6)		
Middle	443 (69.0)	218 (72.9)	192 (70.8)	33 (45.8)		
Low	146 (22.7)	58 (19.4)	53 (19.6)	35 (48.5)		
Family status					29.01	0.001
Single	1881 (79.6)	773 (75.4)	871 (82.2)	237 (84.9)		
Married	459 (19.4)	238 (23.2)	185 (17.5)	36 (12.9)		
Other	24 (1.0)	14 (1.4)	4 (0.4)	6 (2.2)		

^1^ F (2, 2537) → *N* = 642.

**Table 2:** EAT-26 factor loadings based on CFA in a nonclinical Israeli Jewish sample.

Item	Description	Loading
Factor 1: Combined binge–purge and restricting behaviors		
9	I vomit after I have eaten	0.95
20	I feel that others pressure me to eat	0.95
26	I have the impulse to vomit after meals	0.92
8	I feel that others would prefer if I ate more	0.87
2	I avoid eating when I am hungry	0.74
5	I cut my food into small pieces	0.68
24	I like my stomach to be empty	0.68
7	I particularly avoid food with a high carbohydrate content	0.57
15	I take longer than others to eat my meals	0.56
10	I feel extremely guilty after eating	0.55
16	I avoid foods with sugar in them	0.52
4	I have gone on eating binges where I feel that I may not be able to stop	0.44
Factor 2: Dieting behavior and fear and preoccupation with food weight and shape		
11	I am occupied with a desire to be thinner	0.88
1	I am terrified about being overweight	0.81
14	I am preoccupied with the thought of having fat on my body	0.79
23	I engage in dieting behavior	0.78
12	I think about burning up calories when I exercise	0.72
3	I find myself preoccupied with food	0.69
22	I feel uncomfortable after eating sweets	0.61
21	I give too much time and thought to food	0.58
6	I aware of the calorie content of foods that I eat	0.58
17	I eat diet foods	0.43
18	I feel that food controls my life	0.42

**Table 3:** EAT-26 factor loadings based on CFA in a nonclinical Arab Muslim sample.

Item	Description	Loading
Factor 1: Binge–purge, restricting, and dieting behaviors		
17	I eat diet foods	0.73
7	I particularly avoid food with a high carbohydrate content	0.71
16	I avoid foods with sugar in them	0.68
9	I vomit after I have eaten	0.67
23	I engage in dieting behavior	0.60
24	I like my stomach to be empty	0.57
2	I avoid eating when I am hungry	0.57
26	I have the impulse to vomit after meals	0.51
Factor 2: Preoccupation with food, weight, and shape		
12	I think about burning up calories when I exercise	0.74
14	I am preoccupied with the thought of having fat on my body	0.74
11	I am occupied with a desire to be thinner	0.67
1	I am terrified about being overweight	0.55
Factor 3: Fear of loss of control over eating		
3	I find myself preoccupied with food	0.69
21	I give too much time and thought to food	0.66
18	I feel that food controls my life	0.65
4	I have gone on eating binges where I feel that I may not be able to stop	0.57
Factor 4: Eating behavior related to social pressure		
13	Other people think that I am too thin	0.68
8	I feel that others would prefer if I ate more	0.53
20	I feel that others pressure me to eat	0.50

**Table 4:** EAT-26 factor loadings based on CFA in an Arab Christian nonclinical sample.

Item	Description	Loading
Factor 1: Combined binge–purge and dieting behaviors		
9	I vomit after I have eaten	0.99
2	I avoid eating when I am hungry	0.85
24	I like my stomach to be empty	0.76
4	I have gone on eating binges where I feel that I may not be able to stop	0.74
21	I give too much time and thought to food	0.72
23	I engage in dieting behavior	0.70
10	I feel extremely guilty after eating	0.69
20	I feel that others pressure me to eat	0.68
17	I eat diet foods	0.63
22	I feel uncomfortable after eating sweets	0.62
26	I have the impulse to vomit after meals	0.61
16	I avoid foods with sugar in them	0.61
8	I feel that others would prefer if I ate more	0.60
7	I particularly avoid food with a high carbohydrate content	0.58
18	I feel that food controls my life	0.50
Factor 2: Preoccupation with food, weight, and shape		
14	I am preoccupied with the thought of having fat on my body	0.65
11	I am occupied with a desire to be thinner	0.64
12	I think about burning up calories when I exercise	0.62
1	I am terrified about being overweight	0.56
19	I display self-control around food	0.40

## Data Availability

Data sharing is not applicable to this article.
